# Can pelvic radiotherapy induce a leiomyosarcoma of the prostate? (A case report)

**DOI:** 10.11604/pamj.2022.43.56.36997

**Published:** 2022-10-04

**Authors:** Saad Bkiri, Zakaria Tlemsani, Youness Khdach, Karim Bennani, Fayçal Abbad, Mohammed Ghadouane

**Affiliations:** 1 Urology Department, Cheikh Zaid International Hospital, Abulcasis International University, Rabat, Morocco,; 2 Histopathology Department, Cheikh Zaid International Hospital, Abulcasis International University, Rabat, Morocco

**Keywords:** Pelvic radiotherapy, prostate leiomyosarcoma, adenocarcinoma, multimodal treatment, case report

## Abstract

Leiomyosarcoma of the prostate is an extremely rare neoplasm. It represents less than 0.1% of all prostate malignancies. It is considered to have a poor prognosis, an aggressive nature, and high metastatic potential. Additionally, the relationship between radiation exposure for the treatment of primary prostatic cancer and the occurrence of leiomyosarcoma as second cancer at the irradiated site is rare, with unknown etiology. We reported a 72-year-old male known case of prostate adenocarcinoma with radio-hormonotherapy for six years who presented with acute urinary retention. Magnetic resonance imaging revealed a large malignant obstructive prostate with direct invasion of surrounding organs and multiple metastases. Trans-urethral resection of the prostate was performed, and the histopathology result showed high-grade leiomyosarcoma. The patient passed away after four months due to multiorgan failure. In conclusion, there may be a causal relationship between radiation therapy to the prostate and the development of prostate leiomyosarcoma.

## Introduction

Prostatic leiomyosarcoma is a rare tumor that accounts for < 0.1% of all primary prostatic malignancies [1]. Among the sarcomas of the prostate, it represents the most common tumor, accounting for 38-52% of all cases [1,2]. Prostatic leiomyosarcoma is characterized by a very aggressive nature with high metastatic potential [3]. This rare neoplasm develops mainly in prostatic smooth muscle [1]. Until now, the etiology of this uncommon neoplasm is unknown. However, several authors have reported a possible causal effect between pelvic radiotherapy and developing leiomyosarcoma afterward [4,5]. We report an unusual case concerning a patient treated with radio-hormonotherapy for his prostate adenocarcinoma and later developing a leiomyosarcoma of the prostate.

## Patient and observation

**Patient information:** a 72-year-old male presented to the emergency room with acute urinary retention associated with intense diffuse pre-sacral pain radiating to the pubis and the lower extremities. The patient had been diagnosed in 2016 with intermediate-risk prostate adenocarcinoma according to the D'Amico risk classification of prostate cancer. He underwent radiotherapy with a total dose of 76 Gy, targeting the whole prostate and seminal vesicles combined with a long-term hormonotherapy consisting of a 3-month dosage injection of luteinizing hormone-releasing hormone (LH-RH) agonist for two years. No comorbidities or family history of cancer were identified.

**Clinical findings:** the general examination revealed that the patient was agitated, hemodynamically unstable, with a blood pressure of 81/64 mmHg, conscious, and well-oriented. The abdominal examination noticed suprapubic tenderness and bulging. The digital rectal exam showed an asymmetric, enlarged prostate with varied texture and consistency.

**Figure 1:**
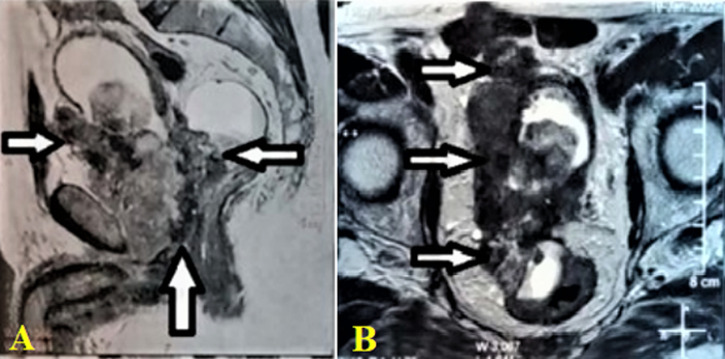
magnetic resonance imaging of the pelvis showing prostatic lesions (arrows) with expansion towards the two seminal vesicles (A); the lower and right lateral wall of the bladder, the rectal wall and the levator ani muscles, the rectal wall, the peri-prostatic bladder fat (B)

**Figure 2:**
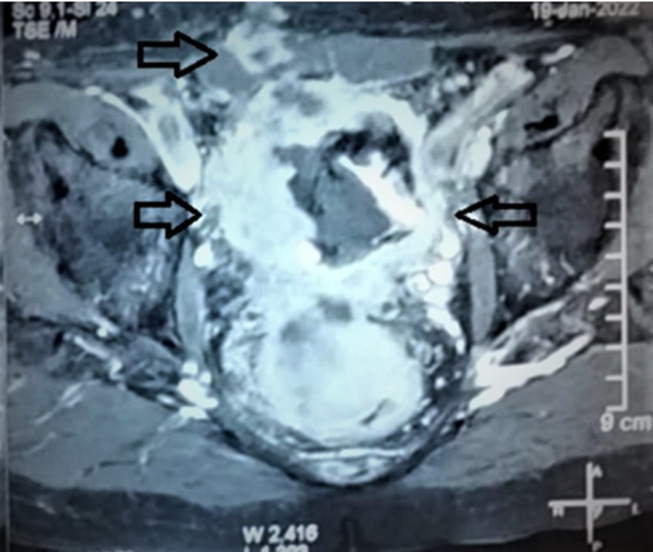
magnetic resonance imaging of the pelvis showing the lesion invading the pubic bone, the right lateral pelvic wall, and the pre-muscular subcutaneous fat (arrows)

**Figure 3:**
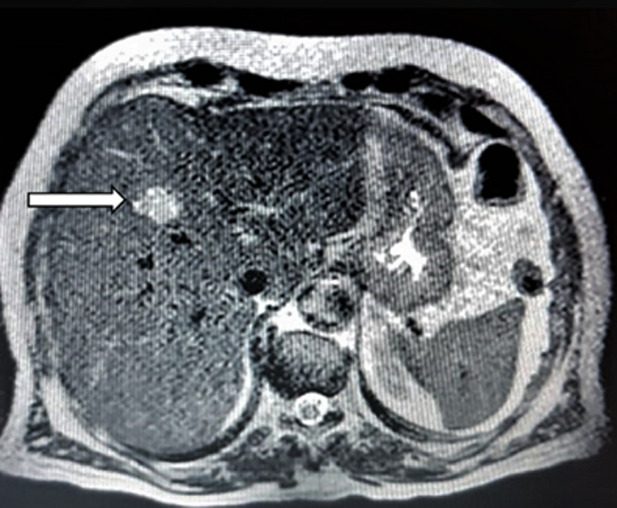
magnetic resonance imaging showing metastatic hepatic lesion 26mm in the IV segment (arrow)

**Figure 4:**
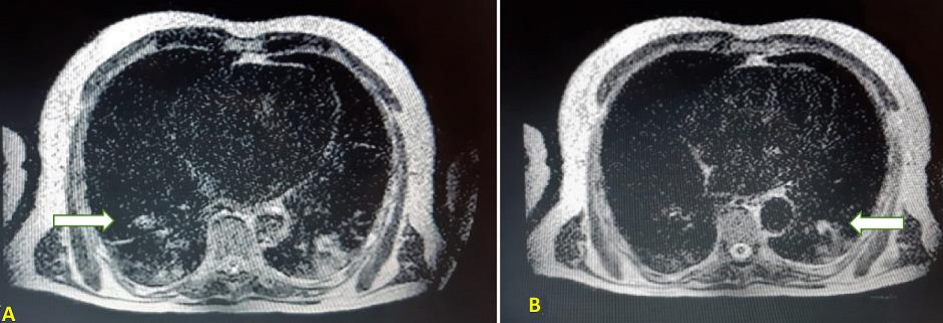
magnetic resonance imaging of the thorax showing multiple pulmonary metastatic lesions; (A) right lobe; (B) left lobe (arrows)

**Diagnostic assessment:** the initial biological check-up showed normocytic normochromic anemia with a hemoglobin level of 6.64 g/dl, acute renal failure was noted with a creatinine level of 19 mg/dL, a hyperkalemia level of 5.4 mmoL/l and an HCO_3_- acidosis level of 15 mmol/l. The cytobacteriological urine test showed a urinary infection with *Escherichia Coli* sensitive only to imipenem. The serum PSA (prostate-specific antigen) level at presentation was 3.98 ng/ml. Magnetic resonance imaging (MRI) was requested immediately, showing a voluminous prostatic tumor process measuring 429mL, locoregional extension towards the seminal vesicles, the mesorectum, the rectum, the levator ani muscles, the rectus muscle, and the pubic bone, with several bilateral internal iliac nodes and some bone lesions in the left iliopubic bone and coccyx (Figure 1, Figure 2). Numerous metastatic hepatic lesions (Figure 3) and pulmonary nodules (Figure 4) were noted too.

**Therapeutic interventions:** firstly, internal foley was inserted. Then, the patient received hydration, a transfusion of 4 red blood cells, an alkalinization with bicarbonate serum, and parenteral antibiotic therapy with intravenous Imipenem 400mg every 6 hours for five days. After a successful clinical and biological recovery, the patient underwent transurethral resection of the prostate.

**Figure 5:**
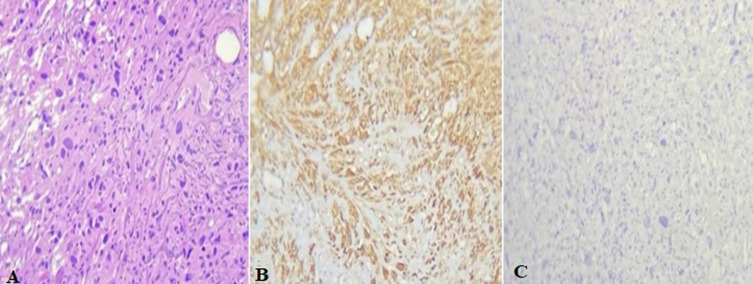
microphotography x20 - haematoxylin eosin - showing; (A) a pleomorphic spindle cell proliferation; immunohistochemical expression showing; (B) H-caldesmon; (C) SMA (smooth muscle actin)

**Follow-up and outcome of interventions:** the patient was discharged in acceptable condition. No complications were noted during or after the surgery. Histopathologic examination of the prostate revealed infiltrative, interlacing fascicles of spindle cells with eosinophilic cytoplasm characterized by high cellularity, marked nuclear atypia, and numerous atypical mitoses. Immunohistochemical analysis demonstrated positive expression of H-caldesmon and smooth muscle actin (SMA) consistent with a high-grade leiomyosarcoma (Figure 5). Following the review of this case in the multidisciplinary consultation meeting, it was decided that the patient is unfit for radical surgery and will only benefit from palliative chemotherapy (consisting of three cycles of Ifosfamide). The patient did not tolerate the 2^nd^ cycle of chemotherapy (severe asthenia and pancytopenia). Unfortunately, the patient passed away after four months due to multiorgan failure.

**Patient perspective:** during treatment, the patient was satisfied with the level of care provided to him. Early with palliative therapy, he remained socially and functionally active. The patient understood the terminal stage of his illness with tremendous support from his family.

**Informed consent:** written informed consent was obtained from the patient family.

## Discussion

Prostate sarcoma is a very rare type of carcinoma that arises from the interstitial tissue of the prostate [6]. This condition generally affects middle-aged patients, but it has also been documented in children and adults aged from 2.5 to 80 years [7]. The most common manifestations are lower urinary tract symptoms, such as urinary urgency, difficulty in urinating, frequent urination, hematuria, and urinary retention [8]. Other associated symptoms include constipation, weight loss, and perineal pain [8]. Our patient fits into the reported age spectrum and presented with suprapubic pain due to acute urinary retention. The lack of early specific symptoms results in more advanced disease at presentation. In fact, up to a third of patients have demonstrable metastases at presentation [8]. The lung is the most frequent metastatic site, followed by the liver [9]. Our patient had metastatic lesions (surrounding tissues, liver, lung) at presentation. The digital rectal exam may identify an enlarged prostate, a hard prostate that has extended into or deformed the capsule, or a large mass extending from the prostate into the rectum, pelvic wall, perineum, and seminal vesicle involvements, or involving the base of the urinary bladder, as seen in our patient [8].

The PSA test may be either normal or elevated, although PSA is insufficient to diagnose or postoperative follow-up of prostatic leiomyosarcoma [10]. The etiology of secondary prostatic leiomyosarcoma remains unknown. However, in some cases, previous pelvic radiotherapy may be related to this condition [3,5,11]. Secondary malignancy after radiation therapy is caused by mutagenesis of soft tissue caused by ionizing radiation. Direct tissue injury is characterized by deoxyribonucleic acid (DNA) breaks, whereas indirect tissue injury is caused by oxidative damage from the free radical formation. Carcinomas and leukemia are more common in areas that receive low doses of radiation or are far from the treatment site. On the other hand, sarcomas are more common in areas exposed to higher doses of radiation or near the treatment site, as seen in our patient [12]. Until now, the possible relationship between pelvic radiotherapy and the occurrence of leiomyosarcoma has still not been proved in scientific literature. This topic is still a permanent debate between the different disciplines. What is very important to consider is that our case is not the only one to be described; several authors reported an association between pelvic radiotherapy and the developing leiomyosarcoma [5,11].

Moreira *et al*. reviewed the possible complications after pelvic radiotherapy. They note that three patients developed prostate cancer after brachytherapy [4]. Furthermore, McKenzie *et al*. described 3 cases of post-irradiation pelvic sarcoma occurring in 8, 15 and 16 years after pelvic radiotherapy for prostate adenocarcinoma [5]. In our case, the patient developed leiomyosarcoma six years after pelvic radiotherapy. More studies are needed to assess clearly the link between pelvic radiotherapy and the development of prostate leiomyosarcoma. If such an association exists, it would be beneficial to closely monitor patients treated with radiotherapy for prostate cancer which appears to be locally controlled. If there is any clinical suspicion, an early biopsy may be needed [4]. Because of the extreme rarity of prostate leiomyosarcoma, specific treatment recommendations have not yet been determined. Multimodal treatment strategies have been employed, including radical surgery, radiotherapy, and chemotherapy [13]. The current approach to managing locally advanced prostate leiomyosarcoma is neoadjuvant radio-chemotherapy or chemotherapy alone. Chemotherapy may contain Ifosfamide, cyclophosphamide, or dacarbazine [14]. Suppiah *et al*. also mentioned that chemotherapy with methotrexate, etoposide, and cisplatin might have a similar result [15]. Our patient was declared unfit for surgery; he received palliative chemotherapy based on Ifosfamide.

Based on the literature, the prognosis of patients with leiomyosarcoma of the prostate is poor [16]. It is reported that 25% of patients already have a metastatic lesion at presentation and that the predictive survival interval is 2 to 5 years after diagnosis in 50-75% of patients [13,14,16]. Our patient passed away after four months of diagnosis due to multiorgan failure.

## Conclusion

Leiomyosarcoma of the prostate is a very rare histological variety with an aggressive nature and a poor prognosis. There may be a causal relationship between radiation therapy to the prostate and the development of leiomyosarcoma, as seen in our patient. If such an association exists, it would be beneficial to closely monitor patients treated with radiation for prostate cancer with biopsy who may appear to be locally controlled. There is no optimal consensus for optimal treatment. However, a multimodality treatment may increase life expectancy compared to a unique treatment.
